# Disseminated Mycobacterium abscessus Infection in a Three-Year-Old Girl With CHARGE Syndrome: A Case Report and Literature Review

**DOI:** 10.7759/cureus.88327

**Published:** 2025-07-19

**Authors:** Yuriko Hiruma, Ryuta Orimoto, Lubna Sato, Yasuaki Tagashira, Kei Takasawa

**Affiliations:** 1 Department of Professional Development, Institute of Science Tokyo Hospital, Tokyo, JPN; 2 Department of Pediatrics and Developmental Biology, Institute of Science Tokyo, Tokyo, JPN; 3 Department of Infection Prevention and Control, Institute of Science Tokyo Hospital, Tokyo, JPN

**Keywords:** charge syndrome, immunocompetent children, multidrug-resistant pathogen, mycobacterium abscessus complex, non-tuberculous mycobacteria

## Abstract

Disseminated *Mycobacterium abscessus* infection is a life-threatening disease that mainly occurs in immunocompromised patients. It is known for its multidrug resistance, and the management for disseminated conditions is not well established. We report a case of severe disseminated *Mycobacterium abscessus* infection in a three-year-old immunocompetent girl with coloboma, heart defect, atresia choanae, retarded growth and development, genital hypoplasia, and ear anomalies/deafness (CHARGE) syndrome. Following microbiologic diagnosis, treatment was started with azithromycin and imipenem/cilastatin, along with exchanges of the intravenous device. However, subsequent deterioration of her condition required the admission to the intensive care unit and finally led to the introduction of a four-agent regimen with azithromycin, amikacin, linezolid, and tigecycline, whose sensitivity was confirmed later. After the initiation of sensitive antimicrobial therapy, she gradually recovered and was discharged with oral azithromycin and sitafloxacin. Immunocompetent pediatric cases of this infection are rare and have been rarely reported. Although her immune function showed no abnormalities, we identified several predisposing factors related to her post-septic condition and her congenital medical history through an investigation of her clinical features with a literature review. While some previous cases of disseminated rapid-growing mycobacterial infections were successfully managed by intravascular device removal alone, this case underscores the need for tailored regimens in pediatric *Mycobacterium abscessus* infections.

## Introduction

*Mycobacterium abscessus* is an environmental bacterium and part of the non-tuberculous mycobacteria (NTM), a group of *Mycobacterium* species excluding *Mycobacterium tuberculosis* complex and *Mycobacterium leprae*. It has various drug resistance mechanisms and is intrinsically resistant to macrolides, aminoglycosides, a wide range of beta-lactams, and fluoroquinolone, which make it difficult to establish effective antibiotic therapy. *M. abscessus* infections were once considered rare and predominantly affect the skin, soft tissue, and respiratory systems. However, it has recently been identified as a pathogen causing disseminated conditions, which is a life-threatening disease with a reported mortality rate of 48% [1]. Disseminated *M. abscessus* infection predominantly occurs in immunocompromised populations, such as patients with HIV/AIDS, malignancies, or transplants [2]. In contrast, it is rare in immunocompetent patients, especially children, due to the low incidence of secondary immunodeficiency. Most pediatric cases are related to cancer, transplants, and immunomodulators [3]. Given the limited number of reported cases to date, the optimal antibiotic regimen for disseminated infections, especially in pediatric patients, remains to be established.

Herein, we report the first case of severe and persistent disseminated *M. abscessus* infection in an immunocompetent three-year-old girl with coloboma, heart defect, atresia choanae, retarded growth and development, genital hypoplasia, and ear anomalies/deafness (CHARGE) syndrome. We reviewed her unique clinical course and background with a literature review to identify treatment strategies for future cases.

## Case presentation

A three-year-old girl was admitted with a fever, vomiting, and hypocalcemia unresponsive to oral medications. She was diagnosed with CHARGE syndrome with CHD7 gene mutation, presenting with bilateral hearing loss, hypoparathyroidism, and developmental retardation. During infancy, she underwent cardiac surgery for aortic coarctation and tracheostomy for laryngomalacia [4].

On admission, the patient was administered cefotaxime due to elevated C-reactive protein (CRP) levels suggestive of bacterial infection (Table 1) and became afebrile. Her serum calcium level was well controlled with intravenous calcium gluconate via a peripherally inserted central catheter (PICC). However, on the ninth day, she developed acute focal bacterial nephritis (AFBN), a condition between pyelonephritis and renal abscess, which was successfully treated with cefepime and vancomycin. On the 17th day of admission, she developed a high fever again. Although repeated seven-day intubated blood cultures were negative, a PICC-related infection was suspected. On the 22nd day, a guidewire-assisted PICC exchange was performed, and the tip culture later revealed *M. abscessus* subsp. *massiliense*. Empiric therapy with azithromycin and imipenem/cilastatin was initiated on the 29th day (Table 1). Subsequent blood cultures, incubated for 10 days, confirmed the presence of the same pathogen in her blood. Microorganisms were identified using a matrix-assisted laser desorption/ionization-time of flight mass spectrometer (MALDI Biotyper®, Bruker, Billerica, MA, USA).

**Table 1:** Laboratory findings AFBN: acute focal bacterial nephritis; ICU: intensive care unit

Laboratory tests	On admission	Day 11 (diagnosed with AFBN)	Day 29 (diagnosed with *M. abscessus* infection)	Day 55 (admission to ICU)	Reference ranges
White blood cells (×10^3^/μL)	12.3	16.1	5.3	3.1	3.3-8.6
Hemoglobin (g/dL)	11.2	7.3	7.8	8.8	11.6-14.8
Platelets (×10^4^/μL)	49.5	18.0	24.0	8.1	15.8-34.8
Albumin (g/dL)	4.1	2.5	2.7	2.2	4.1-5.1
Aspartate transaminase (U/L)	58	47	138	305	13-30
Alanine transaminase (U/L)	45	30	31	49	7-23
Urea nitrogen (mg/dL)	16.9	11.5	5.9	10.9	8.0-20.0
Creatinine (mg/dL)	0.33	0.57	0.24	0.48	0.46-0.79
Sodium (mEq/L)	146	135	141	137	138-145
Potassium (mEq/L)	4.1	3.4	3.5	3.7	3.6-4.8
Calcium (mg/dL)	5.9	6.1	8.5	7.2	8.8-10.1
Phosphorus (mg/dL)	9.1	5.7	4.4	4.3	2.7-4.6
C-reactive protein (mg/dL)	1.13	29.15	6.19	14.24	≤0.14

**Figure 1:**
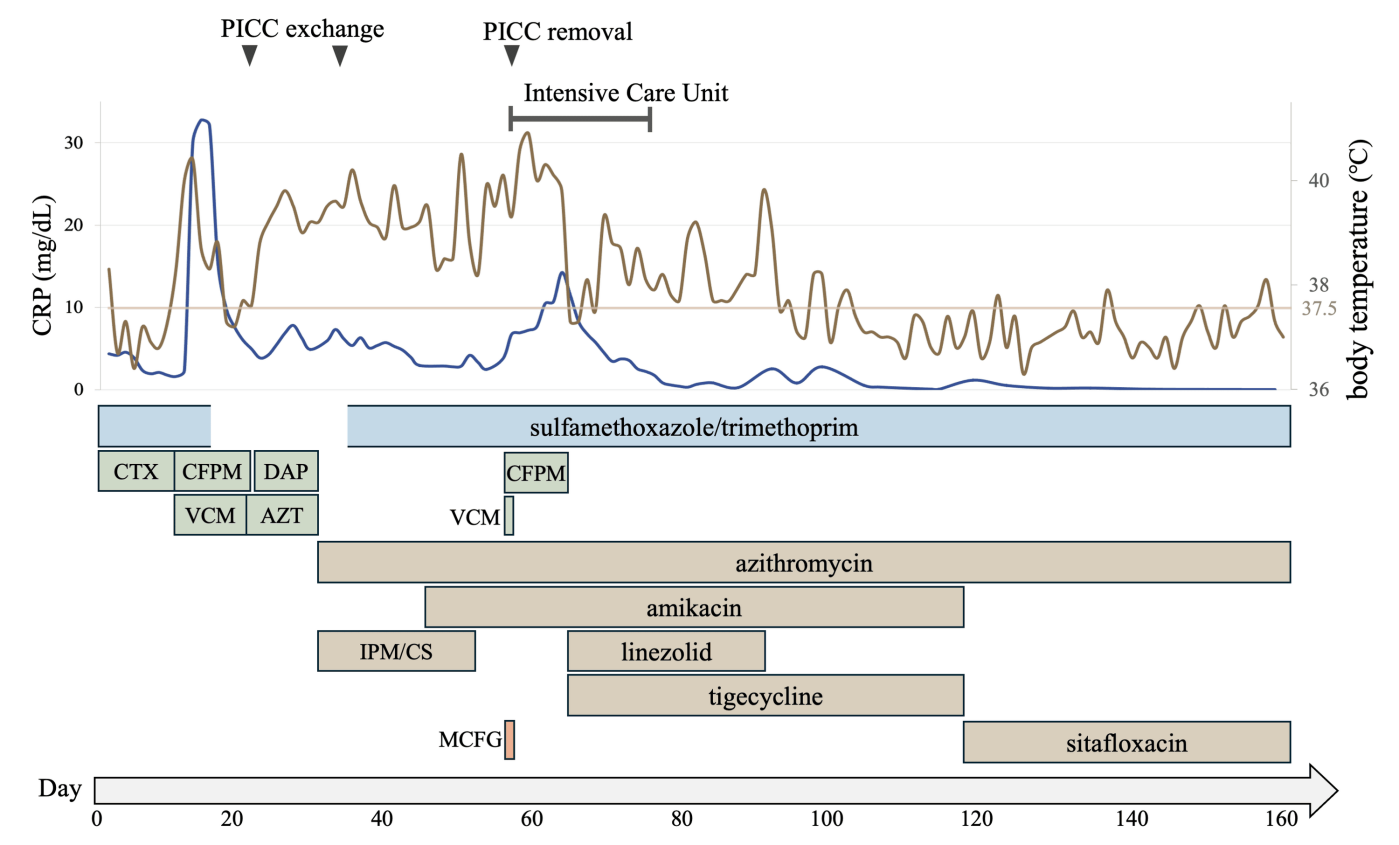
Clinical course with body temperature, CRP levels, and antibiotics On the ninth day of admission, the patient developed a fever, along with elevated CRP levels due to AFBN. After the administration of cefepime and vancomycin, the inflammation declined, and the patient temporarily became afebrile. However, she developed a high fever again on the 17th day, and the CRP level remained elevated. On the 22nd day, we exchanged the PICC, and its tip culture confirmed the diagnosis of *M. abscessus* infection, and azithromycin and imipenem/cilastatin were initiated on the 29th day. We replaced the PICC from a different site on the 35th day. The infection was difficult to control, and the patient stayed in the intensive care unit for 22 days. Finally, following the introduction of linezolid and tigecycline in addition to amikacin, the inflammation gradually subsided. Before discharge, her antibiotics were switched to oral agents. AZT: azithromycin; CFPM: cefepime; CRP: C-reactive protein; CTX: cefotaxime; DAP: daptomycin; IPM/CS: imipenem/cilastatin; MCFG: micafungin; PICC: peripherally inserted central catheter; VCM: vancomycin

Despite the additional PICC exchange, the patient's fever persisted. We added amikacin on the 44th day because the current regimen seemed mismatched to the pathogen, although it had initially been withheld due to ototoxicity concerns. Imipenem/cilastatin was discontinued on the 51st day due to suspected drug-induced anemia and leukopenia. Subsequently, she developed respiratory failure and multiorgan damage with leukopenia of 1,300/μL, thrombocytopenia of 81,000/μL, and elevated aspartate transaminase (AST) of 305 U/L, requiring admission to the intensive care unit (ICU) and intubation on the 55th day (Table 1). A CT scan revealed thrombosis in the left brachiocephalic vein and superior vena cava, presumably formed secondary to calcium infusion via PICC and serving as the infectious focus, along with hepatomegaly indicative of disseminated infection. In addition to the removal of PICC upon ICU admission, linezolid and tigecycline were added to her regimen on the 63rd day to escalate antimicrobial coverage. Subsequently, her condition gradually improved. Linezolid was discontinued due to suspected drug-induced anemia and leukopenia. More than 59 days after the first positive culture of *M. abscessus*, blood cultures for *M. abscessu*s were consistently negative. After 40 days of parenteral four-drug antibiotic therapy following the first negative blood culture, the patient was switched to oral azithromycin and sitafloxacin. She was discharged on the 183rd day with plans for an outpatient antibiotic regimen for 12 months (Figure 1).

Immunological analyses showed no signs of immunodeficiency. The elevated cytokine levels following AFBN were also within normal limits. Additionally, blood cell screening using flow cytometry showed normal lymphocyte counts and distributions at eight months and three years (Table 2). Taken together, she had no apparent signs of immunodeficiency. 

**Table 2:** Immunological features of the patient *: Measurement of lymphocyte aggregation by fluorescence-assisted cell sorting. ^☆^: A specific type of helper T cells. The distribution of lymphocyte subsets, including T cells, B cells, and NK cells, was within normal ranges. Serum immunoglobulin levels were normal. The anti-IFN-γ autoantibody was negative. These findings showed no apparent signs of her immunodeficiency. Anti-IFN-γ: anti-interferon-gamma

	Patient (3 years)	Normal values
T-cell lineages (%)*		
Total T (CD3^+^/lymphocytes)	70.1	60.0-78.0
CD4/CD8 ratio	3.4	1.1-2.5
Total helper T (CD4^+^/CD3^+^)	73.9	53.4-68.0
Helper T, naive (CD45RO^-^CCR7^+^/CD3^+^CD4^+^)	57.7	76.2-93.6
Th1 (CXCR3^+^CCR6^-^/CD3^+^CD4^+^CD45RO^+^CXCR5^-^)	18.6	15.5-34.5
Th2 (CXCR3^-^CCR6^-^/CD3^+^CD4^+^CD45RO^+^CXCR5^-^)	50.8	30.8-52.0
Th17 (CXCR3^-^CCR6^+^/CD3^+^CD4^+^CD45RO^+^CXCR5^-^)	22.7	15.8-28.2
Th1^☆^ (CXCR3^+^CCR6^+^/CD3^+^CD4^+^CD45RO^+^CXCR5^-^)	7.8	2.8-20.4
Tfh (CD45RO^+^CXCR5^+^/CD3^+^CD4^+^)	12.3	1.5-5.0
Total cytotoxic T cells (CD8^+^/CD3^+^)	21.9	23.0-36.4
Cytotoxic T, naive (CD45RO^-^CCR7^+^/CD3^+^CD8^+^)	65.7	24.8-85.5
Cytotoxic T, central memory (CD45RO^+^CCR7^+^/CD3^+^CD8^+^)	6.1	6.5-44.8
Cytotoxic T, effector memory (CD45RO^+^CCR7^-^/CD3^+^CD8^+^)	26.5	4.8-40.0
Cytotoxic T, T_EMRA_ (CD45RO^-^CCR7^-^/CD3^+^CD8^+^)	1.7	3.0-33.9
B-cell lineages (%)*		
B cells (CD19^+^/lymphocytes)	22.9	8.7-23.5
Memory B cells (CD27^+^/CD19^+^)	12.3	9.3-24.5
NK-cell lineage (%)*		
NK cells (CD3^-^CD16^+^CD56^+^/lymphocytes)	4.6	2.3-15.3
Immunoglobulin levels (mg/dL)		
IgG	922	640-1,500
IgA	166	50-250
IgM	65	30-220
Anti-IFN-γ autoantibody	Negative	Negative

## Discussion

Pediatric cases of *M. abscessus* infections are rare, with most patients being immunosuppressed due to cancers or transplants [3]. Primary immunodeficiencies that predispose children to NTM infections include defects in cellular immunity and interferon-gamma pathways, such as Mendelian susceptibility to mycobacterial diseases, a rare monogenic defect affecting interferon-gamma function [3,5]. However, our patient had no apparent signs of these immune characteristics. Overall, we estimated that the patient had several factors that made her vulnerable to this pathogen.

First, CHARGE syndrome has substantially influenced her complicated clinical course. Immunodeficiency is a feature of CHARGE syndrome, and it clinically overlaps DiGeorge syndrome (DGS) [6]. Hoyos-Bachiloglu et al. reported a case of disseminated NTM infection in a patient with partial DGS presenting with normal T-cell function without thymic hypoplasia, which is a condition similar to our case [7]. Despite no apparent abnormalities in immunological analysis, an undetected immune dysfunction associated with CHARGE syndrome may have contributed to this infection. Additionally, high-concentration calcium intravenous infusion via a PICC to maintain serum calcium levels for hypoparathyroidism, a complication of CHARGE syndrome, may lead to calcified venous thrombus formation, providing an intravascular focal infectious site and contributing to the refractory clinical course. Furthermore, frequent medical procedures, such as tracheostomy suctioning, increase exposure to moist environments. Therefore, we hypothesized that congenital disorders contribute to potential vulnerabilities to opportunistic infections. As with our literature review of immunocompetent pediatric cases of disseminated rapidly growing mycobacterium infections (Table 3), these immunocompetent patients commonly involve congenital diseases with systematic presentations, which may support our hypothesis.　

**Table 3:** Disseminated rapidly growing mycobacterium infections in immunocompetent children CHD: congenital heart disease; GDD: global developmental delay; CVC: central venous catheter

Author (ref.)	Age, sex	Focal infection sites	Remarkable health condition	Diagnostic exam	Pathogen
Martin et al. [8]	16-month-old, F	Central nervous system	Neurofibromatosis type 1	Brain biopsy	M. abscessus
Jamal et al. [9]	16-month-old, M	Bloodstream, respiratory system	CHD, congenital anomaly, hypothyroidism, chronic lung disease (ventilator dependent), GDD	Blood culture, tracheal secretion culture	M. abscessus
Turock et al. [10]	14-year-old, M	Bloodstream	Hemophilia B	Blood culture, catheter tip culture	*M. cosmeticum*
Knight and Naik [11]	2-year-old, M	Bloodstream	Type 3 intestinal failure, long-term home parenteral nutrition via CVC	Blood culture	M. mucogenicum

Moreover, preceding AFBN may have contributed to the development of this opportunistic infection by placing the patient in a post-septic immunosuppressed state. Similarly, a case of *M. abscessus* bloodstream infection in an immunocompetent adult following septic pneumonia has been reported [12].

The management of disseminated *M. abscessus* infections remains uncertain and challenging because of multidrug resistance and the need for prolonged therapy periods. Amikacin, clarithromycin, and cefoxitin have shown high susceptibility and low resistance rates, followed by linezolid and imipenem [2,13]. Although no established antibiotic regimen exists for *M. abscessus* infections in children, antibiotic combination therapy is required and should be adjusted based on the activity of antimicrobial agents [3]. Conversely, among immunocompetent patients, there are many reports of significant recovery from rapidly growing mycobacterium bloodstream infections, including *M. abscessus*, after PICC removal without antibiotics [14]. In our case, we exchanged the PICC and initiated treatment with a combination of azithromycin and imipenem/cilastatin, avoiding amikacin due to ototoxicity concerns. However, the infection was not controlled, and the subsequent removal of the intravenous device and the introduction of amikacin, linezolid, and tigecycline resulted in gradual improvement. Compared to previous cases, we initiated therapy with a narrower selection of safer agents, which require additional antibiotics later due to clinical deterioration. In severe cases, empiric antibiotics covering a broad range of resistance patterns may need to be prioritized over the risk of adverse effects. The sensitivity testing later revealed susceptibility to amikacin (minimum inhibitory concentration (MIC) 8 μg/mL) and linezolid (MIC ≤1 μg/mL), which confirmed the new regimen's effectiveness. The limited evidence about regimens for pediatric cases, their multiple adverse effects, and the prolonged duration of wait for sensitivity test results contributed to the challenges of selecting optimal antibiotics. Furthermore, the PICC exchanges did not significantly improve the infection, likely due to thrombus formation predisposed by hypothyroidism treatment.

The limited number of reported cases with the variability in microbial resistance profiles and antibiotic-related adverse effects makes it difficult to standardize the management of disseminated *M. abscessus* infection in children. Further exploration for effective therapeutic and preventive strategies and early detective methods may be warranted for future cases.

## Conclusions

We encountered a case of disseminated *M. abscessus* infection in an immunocompetent child. The development of this rare and life-threatening condition may be influenced by multiple factors, including congenital diseases and post-septic status. Although its management strategies remain inconclusive, especially in pediatric and immunocompetent cases, we suggest initiating empiric antimicrobial therapy, prioritizing including effective agents over potential side effects in severe cases.
